# Seroprevalence and Associated Risk Factors of Rift Valley Fever in Livestock from Three Ecological Zones of Malawi

**DOI:** 10.3390/pathogens11111349

**Published:** 2022-11-14

**Authors:** Henson Kainga, Marvin Collen Phonera, Elisha Chatanga, Simegnew Adugna Kallu, Prudence Mpundu, Mulemba Samutela, Herman Moses Chambaro, Masahiro Kajihara, Doreen Mainza Shempela, Jay Sikalima, Walter Muleya, Misheck Shawa, Julius Chulu, Gilson Njunga, Martin Simuunza, Ayato Takada, Hirofumi Sawa, Edgar Simulundu, Ngonda Saasa

**Affiliations:** 1 Department of Veterinary Epidemiology and Public Health, Faculty of Veterinary Medicine, Lilongwe University of Agriculture and Natural Resources, Lilongwe 207203, Malawi; 2 Department of Disease Control, School of Veterinary Medicine, University of Zambia, Lusaka 10101, Zambia; 3 Department of Animal Health and Livestock Development, Ministry of Agriculture, Lilongwe 207203, Malawi; 4 Department of Veterinary Pathobiology, Faculty of Veterinary Medicine, Lilongwe University of Agriculture and Natural Resources, Lilongwe 207203, Malawi; 5 College of Veterinary Medicine, Haramaya University, Dire Dawa P.O. Box 138, Ethiopia; 6 Department of Environmental and Occupational Health, Levy Mwanawasa Medical University, Lusaka 33991, Zambia; 7 Department of Paraclinical Studies, School of Veterinary Medicine, University of Zambia, Lusaka 10101, Zambia; 8 Department of Biomedical Sciences, School of Health Sciences, University of Zambia, Lusaka 10101, Zambia; 9 Virology Unit, Central Veterinary Research Institute (CVRI), Ministry of Fisheries and Livestock, Lusaka 10101, Zambia; 10 Division of Global Epidemiology, International Institute for Zoonosis Control, Hokkaido University, Sapporo 001-0020, Japan; 11 Churches Health Association of Zambia, Lusaka 10101, Zambia; 12 Department of Biomedical Sciences, School of Veterinary Medicine, University of Zambia, Lusaka 10101, Zambia; 13 Division of Collaboration and Education, International Institute for Zoonosis Control, Hokkaido University, Sapporo 001-0020, Japan; 14 Japan Division of International Research Promotion, International Institute for Zoonosis Control, Hokkaido University, Sapporo 001-0020, Japan; 15 Japan Global Virus Network, Baltimore, ML 21201, USA; 16 One Health Research Center, Hokkaido University, Sapporo 001-0020, Japan; 17 Macha Research Trust, Choma 20100, Zambia

**Keywords:** Rift Valley fever, seroprevalence, IgG, IgM, ELISA, livestock, risk factors, Malawi

## Abstract

The epidemiology of Rift Valley fever (RVF) is poorly understood in Malawi. Here, a cross-sectional study was conducted (March–June 2020) to investigate the seroprevalence and potential risk factors of RVF virus (RVFV) in cattle, goats, and sheep in three ecological zones of Malawi. A total of 1523 serum samples were tested for anti-RVFV IgG and IgM antibodies by ELISA. Additionally, a questionnaire survey was used to assess potential RVF risk factors. The overall seroprevalence was 17.14% (261/1523; 95% CI = 15.33–19.11) for individual livestock and 33.24% (120/361; 95% CI = 28.18–38.11) for the livestock herd. Seroprevalence was significantly high in sheep (25.68%, 95% CI = 19.31–33.26) compared with cattle (21.35%, 95% CI = 18.74–24.22) and goats (7.72%, 95% CI = 5.72–10.34), (*p* = 0.047). At the individual livestock level, the risk was elevated in female livestock (OR: 1.74, 95% CI = 1.08–12.82) (*p* = 0.016), while at the herd level, areas receiving approximately 1001–1500 mm of rainfall (OR: 2.47, 95% CI = 1.14–5.37) (*p* = 0.022), areas of rainfall amount greater than approximately 1600 mm (OR: 2.239, 95% CI = 1.07–8.82) (*p* = 0.023), and mixed species herds (OR: 10.410, 95% CI = 3.04–35.59) (*p* = 0.001), were significant risk factors. The detection of IgM antibodies confirmed active circulation of RVFV in Malawi. Therefore, monitoring of RVF in animals, humans, and vectors using a “One Health” approach, along with community sensitization among the high-risk populations, could help mitigate the threat posed by this zoonotic disease in Malawi.

## 1. Introduction

Rift Valley fever (RVF) is an emerging arthropod-borne viral zoonotic hemorrhagic fever caused by RVF virus (RVFV), a member of the *Phlebovirus* genus, family *Phenuiviridae*, within the order *Bunyavirales* [1]. The RVFV genome is organized in three negative-sense, single-stranded RNA segments, namely large (L), medium (M), and small (S). The large segment encodes the RNA-dependent RNA polymerase [2]; the M segment encodes envelope glycoproteins, Gn and Gc, plus two accessory proteins, NSm and the 78-kDa protein [3]. The S ambisense segment encodes for nucleoprotein (NP; 27 kDa) and non-structural protein (NSs; 31-kDa). RVFV is divided into 15 major genetic lineages (A-O), based on S, M, and L segments, despite having over 33 strains in circulation [4,5,6]. Juma et al. [7] reported a total of 234 sequences of RVFV, which are correctly classified at the phylogenetic level [8].

The RVFV has been detected throughout sub-Saharan Africa since 1931 [9,10,11,12]. Even though RVF is endemic to sub-Saharan Africa, disease outbreaks have been reported in Egypt, the Arabian Peninsula, Comoros, Mayotte, and Madagascar [10,11,12,13]. Primarily, RVF affects domestic and wild ruminants; however, disease spill-over to humans is occasionally reported. Recent human RVF outbreaks were reported in Kenya in 2020 [14] and outside the African continent in the French territory of Mayotte [15]. The spread of RVFV outside the endemic region raises concern regarding the threat of virus introduction to new geographical areas [16].

Rift Valley fever usually manifests as explosive epizootics with prolonged inter-epidemic periods (IEPs) of approximately 8 to 15 years [17]. The occurrence of RVF in the sub-Saharan African region is not limited to epizootic periods but also occurs during IEPs [18,19,20,21,22]. The viral maintenance during IEPs is mainly accomplished by mosquito species of *Aedes* (*Neomelaniconion*) *mcintoshi* and *Aedimorphus* [23,24,25]. Infected mosquitoes lay eggs at the edges of dambos; they hatch during periods of anomalous rainfall and initiate disease transmission [25,26,27]. The recruitment of secondary bridge mosquitoes such as *Culex*, *Anopheles*, and *Aedes* [25,26,27] results in wide-spread infection. The low level of detection of the RVFV in mosquitoes coupled with the high seroprevalence rates in domestic and wild ruminants during the IEP suggests a wider role of angulates in the epidemiology of RVF [28]. The RVFV transmission to humans is predominantly by direct contact with infected animals and body fluids. Nevertheless, to a lesser extent, mosquito bites indirectly transmit RVFV to humans [29].

In many areas where RVF is endemic, the IEP is mostly followed by outbreaks either in livestock or humans soon after heavy rainfall [30,31]. Many RVF outbreaks reported in livestock are associated with heavy rainfall and flooding and cause high mortality in young animals and/or abortions in pregnant animals [32,33]. Several other economic burdens, including reduced productivity and international livestock trade restrictions, were reported [34,35,36]. For example, the 2006–2007 RVF outbreak in Tanzania caused economic losses ranging from USD 352,750.00 to USD 4,243,250.00, attributed to decreased productivity, livestock mortality, and suspension of international trade in livestock and livestock products [34,35,36]. Subsequent to the huge adverse impact of the outbreaks, some East African countries developed policies for preparedness and control measures against RVFV [37]. Furthermore, these countries described potential risk factors associated with RVFV seropositivity of animals and humans that varied with the time at which the study was conducted (outbreak or IEP) [38,39]. The common risk factors associated with RVFV seropositivity for humans and animals include heavy rainfall, vegetation density, topography, large water bodies, land use, drainage, temperature, age, and mosquito abundance [38,39,40,41,42].

Information on the current status of RVF in Malawi remains speculative [43]. The last report on RVF in Malawi was made in 1992, when a seroprevalence of 18.1% was reported in cattle [44]. Since then, there have been no reports of RVFV circulation in cattle, goats, or sheep. For the last five years of this period, RVF was reported in Mozambique, Tanzania, and Zambia [18,19,22]. In these reports, the evidence of the possible circulation of RVFV was detected from serum samples obtained from susceptible hosts, including those showing no clinical signs [18,19,22,31,33]. Of note, Malawi’s neighboring countries reported higher frequencies of RVFV circulation in their respective countries. Of special concern is the increased livestock movement between Malawi and the neighboring countries and lack of awareness among livestock farmers in Malawi [43]. In addition, there are no surveillance, control, or prevention measures such as routine vaccination in place for the same [43]. This situation hinders generation of important epidemiological baseline data for RVF. Lack of recent epidemiological data on RVF in Malawi makes it difficult to design evidence-based preventive and control strategies, as those demonstrated in Kenya [37]. Furthermore, the lack of information on such an important zoonosis is detrimental to the efforts of safeguarding public health and livestock development. Thus, the current study was conducted to provide evidence of RVFV circulation, identify the associated risk factors, and update our current knowledge on the epidemiology of RVF in livestock in Malawi.

## 2. Materials and Methods

### 2.1. Study Sites

**Figure 1:**
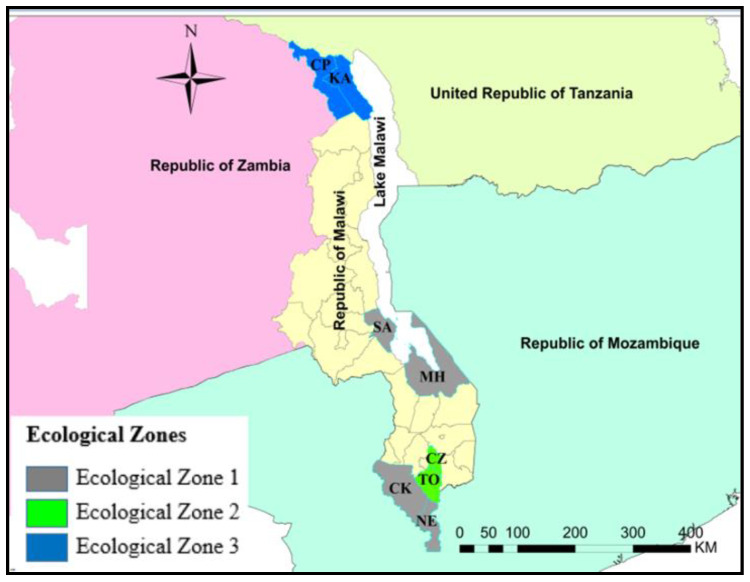
Map of Malawi showing the ecological zones and districts. CP = Chitipa, KA = Karonga, SA = Salima, MH = Mangochi, CZ = Chirazulu, TO = Thyolo, CK = Chikwawa, and NE = Nsanje.

Malawi is a landlocked and agriculture-based country covering 118, 484 square kilometers in southeast Africa, situated within latitudes 9° and 18° S, and longitudes 32° to 36° E. Malawi shares borders with Tanzania to the north, Mozambique to the southeast and southwest, and Zambia to the west. The study was conducted in eight districts selected from all three ecological zones of Malawi, as shown in (Figure 1). The eight districts were purposively selected to include different annual rainfall patterns, vegetation, and livestock density. The districts were Chitipa (CP), Karonga (KA), Salima (SA), Mangochi (MH), Chiradzulu (CZ), Thyolo (TO), Chikwawa (CK), and Nsanje (NE). CP and KA are located in the northern part of Malawi along Songwe River bordering Tanzania and Zambia. SA in the central part of Malawi and MH in the southern part of Malawi are located along the shores of Lake Malawi with characteristic wide range of dambo areas and dense vegetation cover; CZ and TO in the southern part of Malawi are located adjacent to Zomba district where RVF was previously reported [44,45]. CK and NE are on the southernmost part of Malawi along the Shire River valley bordering Mozambique. These two districts have high ruminant population and experience frequent flooding.

### 2.2. Agro-Ecological Zones in Malawi

The majority of livestock activities are conducted under extensive management systems in all three agro-ecological zones. Ecological Zone 1 (EZ1) covers low lands of semi-arid areas found mainly on the shores of Lake Malawi and in the Rift valley areas of the Shire valley such as the areas of Salima, Mangochi, Chikwawa, and Nsanje districts. EZ1 lies 500–1000 m above sea level and receives an annual rainfall of less than 1000 mm. Ecological Zone 2 (EZ2) covers the highland plains of the Shire highlands, Lilongwe, Kasungu, and Mzimba, lying 1000–1500 m above sea level and includes Thyolo and Chiradzulu districts. This zone receives an annual rainfall of 1000–1500 mm. Ecological Zone 3 (EZ3) covers high altitude areas of the Vipya and Nyika plateaus and the Chitipa and Karonga districts, lying more than 1500 m above sea level. It has a total annual rainfall of more than 1500 mm. A high proportion of this zone encompasses forest reserves and national parks. There are approximately two million smallholder families and 30,000 estates in Malawi [46]. Most family-operated smallholdings depend on subsistence farming based on mixed crop and livestock farming [46,47].

### 2.3. Study Design and Sampling

Two veterinary stations were identified at each District Agriculture Office (DAO). An average of 5 livestock (cattle, goats, and sheep) per farm was assumed. Farmers were selected using a systematic random sampling technique from an updated livestock census book based on sample size proportionally distributed to the districts. At the herd level, all livestock were sampled if the herd size was < 7, and a maximum of 15 was sampled if the herd size was > 7, as previously described [46,48,49,50]. Assistant veterinary officers and lead farmers were consulted to identify the livestock owner and herd size after selecting from the livestock census book. Since individual animals could not be identified within herds, arbitrary numbers were assigned to individual animals within a herd which was later used for simple random selection by a raffle drawing. The number of livestock herds were obtained by counting the number of herds included in the study [51].

### 2.4. Sample Size Estimate

The sample size was estimated using Cochran’s Formula [52]. The assumption was that the population was very large and heterogenous among the ecological zones. The calculated sample size was 384. However, RVF is a less contagious disease among livestock; thus, in order to increase the precision of study estimates and obtain similar accuracy to that of simple random sampling for districts, the sample size was recalculated. The study expected at least five (5) livestock to be available in each sampled veterinary station, and as such, the average rate of RVF homogeneity (*p*) was estimated to be 0.156, and recalculated as:

N_new_ = *n* [1 + *p ×* (*m* − 1)] (1)

where *n* = 384, *p* = 0.156, and *m* = 5, representing the average number of animals to be sampled from each veterinary station. The new sample size was 2220 livestock.

### 2.5. Sample Collection, Storage, and Transportation

The total sample size was proportionally divided into ecological zones (EZ) and district zones based on total district livestock population. The livestock population for districts and total livestock herds were obtained from Malawi national agricultural production estimates (APES), livestock census for first round conducted in January 2019. The livestock were selected in the ratio 3:2:1 for cattle, goats, and sheep, respectively [53].

Whole blood was aseptically collected from cattle, goats, and sheep of all eligible age groups by external jugular or coccygeal venipuncture. One set of whole blood samples were collected in plain vacutainer tubes for serum, and another set was collected in EDTA vacutainer tubes (5 mL in each). Serum was separated from whole blood by centrifugation at 1000× *g* for 15 min as per World Organization for Animal Health (WOAH) protocol [54] and later aliquoted into two milliliter Eppendorf tubes. The samples were immediately stored at −80 °C at the African Union Center of Excellence for Tick and Tick-borne Diseases (AU-CTTBD). Thereafter, the samples were triple-packed and transported by road, in a frozen state, to the Department of Disease Control laboratories at the Samora Machel School of Veterinary Medicine at the University of Zambia (UNZA), where they were stored at −80 °C in readiness for processing and analysis.

### 2.6. Serum Sample Laboratory Analysis

#### 2.6.1. IgG ELISA

All serum samples were tested for the presence of RVFV antibodies. ID Screen^®^ RVF competition multi-species indirect kit (ID- Vet Innovative, Grabels, France) was used to investigate the presence of IgG following the manufacturer’s instruction. A suspect or negative (S/N) ≤ 40% was considered as positive, otherwise negative. All samples were run in duplicate, and the test was valid if the mean value of the positive control optical density (OD_PC_) was less than 30% of the negative control (OD_NC_), given as OD_PC_/OD_NC_ < 0.3 and if the mean value of the negative control optical density (OD_NC_) was greater than 0.7, given as OD_NC_ > 0.7. All the runs were valid on both criteria. All doubtful *S*/*p*% values were considered negative. The reported diagnostic sensitivity and specificity for the kit were 98% and 100%, respectively [55,56].

#### 2.6.2. IgM ELISA

All samples were run in duplicate for IgM investigation, which indicate recent infection [55,57], using ID Screen^®^ RVF IgM Capture Multi-species direct kit (IDvet Innovative, Diagnostics, Garbles, France), as per manufacturer’s instruction. The test was valid if the mean optical density of the positive control (OD_pc_) was greater than 0.350, given as net OD_pc_ > 0.350 and the ratio of the mean OD_pc_ to mean optical density for negative control (OD_nc_) was greater than three, given as (net OD_pc_/|net OD_nc_|) > 3. Samples with suspect or positive (*S*/*p*) ≤ 40% were considered negative, and samples with *S/p* ≥ 50% were considered positive and anything <50% is negative.

### 2.7. Questionnaire Administration

A structured questionnaire was used to capture information on potential risk factors for individual and herd level seropositivity to RVFV. The questionnaire, firstly, gathered demographics information of herd owners; secondly, information on livestock species, herd dynamics (herd size: small < 25, medium 26–50, and large > 51) [58], age groups (generated according to degree of physiological activities and interactions and livestock management), source of livestock (within or outside the district), cross-border livestock interaction (present or absent), grazing system (stall feeding or grazing), type of grazing land (private grazing grounds or communal grazing grounds), grazing type (mixed species or single species), and presence of livestock market was collected. The third part gathered information about herd owners’ knowledge of RVF, its causative agent, clinical signs, occurrence of abortion, neonatal death, mode of transmission, knowledge of *Aedes* spp. of mosquito, and knowledge of zoonotic nature of RVF. Lastly, we gathered information about ecological factors, such as occurrence of heavy rainfall, flooding, occurrence of mosquito, presence of permanent water points, and degree of vegetative cover. Selected herd owners who did not want to participate in the study were replaced by other herd owners and corresponding herds within the veterinary stations.

### 2.8. Data Analysis

All data were entered, cleaned, and validated in a Microsoft Office™ Excel^®^ 2019 spreadsheet. The RVFV ELISA test result (positive or negative) was the dependent variable in this study. Descriptive and inferential analyses were performed in IBM SPSS version 20 (IBM Corp) and Microsoft Office™ Excel^®^ 2019 spreadsheets. One-way ANOVA and Student’s *t*-test were used to test mean differences of variables. Bivariate analysis was performed using the Pearson Chi-Square test of association (and Fisher’s exact test, where appropriate) at a moderate significance of *p* ≤ 0.250. Univariable linear regression models with the expected outcome RVFV ELISA test results, were generated to check multicollinearity. Thereafter, a multivariable linear regression model was fitted, which included variables that retained significance (*p* < 0.05) in the univariable linear regression analysis [20,59]. The generated multivariable model was tested for goodness of fit and predictability using the Hosmer–Lemeshow test and Omnibus test, respectively. The potential risk factors had *p*–values less than 0.05.

## 3. Results

### 3.1. Description of Study Population

The study recruited 361 livestock farmers who gave written consent for questionnaire administration and whole blood sample collection from their livestock. Of the 361 participants, 58.17% (210/361) were male, and 41.83% (151/361) were female. Of the 361 participants, 98.06% (354/361) depend on subsistence farming for their livelihoods, while 1.94% (7/361) had other income-generating activities. The species composition at herd level was 62.05% (224/361), 34.07% (123/361), and 3.88% (14/361) for cattle, goat, and sheep, respectively. In this study, herd size distribution varied, with 84.49% (305/361) from small herds (≤25 animals), 4.98% (18/361) from medium herds (26–50 animals), and 10.53% (38/361) from large herds (>50 animals). The sampling for sheep flocks was not as expected because some farmers refused due to fear of livestock death caused by unknowns in CP, TO, and CZ districts.

**Figure 2:**
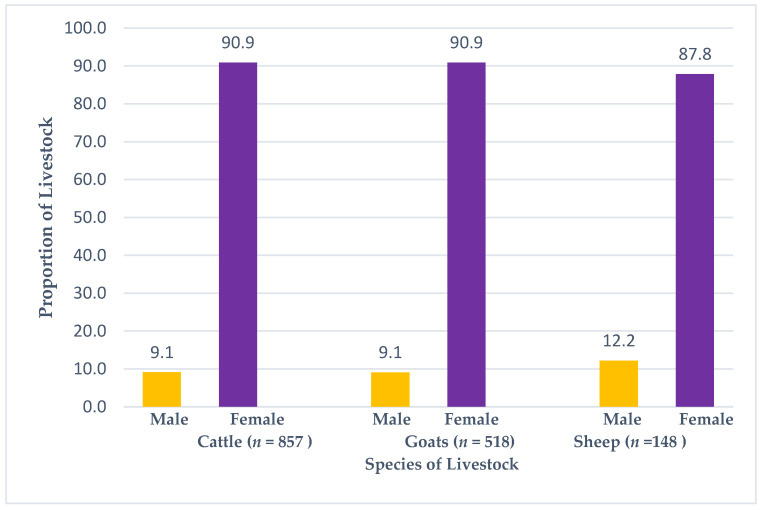
Proportion of sampled animals by livestock species and sex.

The study site comprised three ecological zones (EZ); EZ 1 consisted of four districts, namely Salima (SA), Mangochi (MH), Chikwawa (CK), and Nsanje (NE); EZ2 had two districts, Thyolo (TO) and Chiradzulu (CZ); and lastly, EZ3 had two districts, Chitipa (CP) and Karonga (KA) (Figure 1). A total of 1523 livestock whole blood samples were collected from cattle 56.27% (857/1523), goat 34.01% (518/1523), and sheep 9.72% (148/1523) (Table S1) (Supplementary Materials). Among these, 90.54% (1379/1523) were female, and 9.46% (144/1523) were male. The proportions of the female cattle, goats, and sheep were 90.90% (779/857), 90.93% (471/518), and 87.84% (130/148), respectively (Figure 2). The age of cattle was divided into four categories: young < 2 years old was 24.85% (213/857), sub-adults 2–4 years old was 29.87% (256/857), adults 5–8 years was 40.61% (348/857), and old-age group for those above 9 years was 4.66% (40/857). Similarly, the ages of goats were divided into four categories: < 2 years old was 30.50% (158/518), 2–3 years was 40.54% (210/518), 4–5 years was 26.26% (136/518), and those above 6 years was 2.70% (14/518). The ages for sheep were also in four age categories: < 2 years old was 31.08% (46/148), 2–3 years was 45.95% (68/148), 4–5 years was 21.62% (32/148), and those above 6 years was 1.35% (2/148).

### 3.2. RVFV Antibodies Results

The study screened 1523 serum samples for antibodies against RVFV and the optical density readings (OD) used for RVFV seropositivity determination of livestock are presented in Tables S1 and S2 (Supplementary Materials). The total reactors per species were higher in cattle than in goats and sheep at 183, 40, and 38, respectively (*p* = 0.016). The distribution of reactors was: 169 for EZ1, 37 for EZ2, and 55 for EZ3 (Table S3).

### 3.3. Seroprevalence by Livestock Species at Individual Animal Level

The overall combined seroprevalence was 17.14% (261/1523, 95% CI = 15.33–19.11), although this was significantly high (*p* = 0.047) in sheep (25.68%, 95% CI = 19.31–33.26) compared with cattle (21.35%, 95% CI = 18.74–24.22) and goats (7.72%, 95% CI = 5.72–10.34). The IgG overall seroprevalence was 14.18% (216/1523, 95% CI = 12.49–16.06) and 2.95% (45/1523, 95% CI = 2.19–3.97) for IgM (Table 1).

### 3.4. Seroprevalence in Ecological Zones and Districts

The seroprevalence varied across ecological zones and districts. EZ1 had the highest seroprevalence (20.34%, 169/831) compared with EZ3 (14.55%, 55/378) and EZ2 (11.78%, 37/314) (*p* = 0.022) (Table 2). District seroprevalence ranged from 6.22% to 25.68%. The highest seroprevalence was observed in the Salima District (23.76%, 95% CI = 18.19–30.35), with the lowest in Karonga District (6.22%, 95 CI = 3.59–10.55) (Table 2).

### 3.5. Seroprevalence According to the Sex and Age

The overall seroprevalence appeared to be higher in male livestock than in female counterparts (Table 3). Similarly, males had higher IgG seroprevalence than female livestock (*p* = 0.031). Furthermore, the overall seroprevalence across the age groups was higher in sub-adults (2–4 and 2–3 age groups) for cattle and goats (*p* = 0.023 and *p* = 0.046, respectively), while old-age (≥6) had higher seroprevalence in sheep (*p* = 0.029) (Table 4).

### 3.6. RVFV Seroprevalence at Livestock Herd Level

The study recruited 361 livestock herds of which 62.05% (224/361, 95% CI = 56.80–67.04), 34.07% (123/361, 95% CI = 29.24–39.25) and 3.88% (14/361, 95% CI = 2.21–6.57) were cattle, goat, and sheep, respectively. Livestock herds were IgG-positive in all sampled districts, unlike the IgM antibodies which were observed in only five out of eight districts (Table S4).

The overall herd seroprevalence was 33.24% (120/361, 95% CI = 28.18–38.11). All (100%) the sheep herds sampled were seropositive, of which 64.29% (9/14) were IgG seroprevalent and 35.71% (5/14) were IgM seroprevalent. The herd seroprevalence for cattle was 36.16% (81/224, 95% CI = 29.51–42.41), of which 77.78% (63/81) were seroprevalent for IgG and 22.22% (18/81) were seroprevalent for IgM. The herd seroprevalence for goats was 20.33% (25/123, 95% CI = 13.82–28.73) of which 68.00% (17/25) were seroprevalent for IgG and 32.00% (8/25) were seroprevalent for IgM.

We also compared the herd level seroprevalence across the different ecological zones (EZ). The herd seroprevalence for EZ2 was higher at 36.36% (52/143, 95% CI = 28.61–44.55), followed by EZ1 34.12% (29/85, 95% CI = 24.40–45.88), then EZ3 with 29.32% (39/133, 95% CI = 21.92–37.95).

Furthermore, the overall herd seroprevalence (combined IgG and IgM) at district level had a median of 43.93% (range 14.37–84.25%, (*n* = 8)) (Table 5). Mangochi and Nsanje districts had overall herd seroprevalence of more than 75%, while Chiradzulu and Karonga districts had 16.13% and 14.33%, respectively. The herd seroprevalence varied among the three species such that sheep had higher seroprevalence of 100% (*n* = 5) compared with cattle and goats (Table 5).

### 3.7. Analysis of Association between Potential Risk Factors and RVFV Seropositivity

Frequencies and proportions of the epidemiological factors varied across the study area (Table 6 and Table 7). It was found that 46.67% (7/15) and 40.00% (6/15) of the variables were significant for individual livestock and herds (*p* = 0.05), respectively. Thereafter, the variables were screened for multicollinearity using univariate linear regression (Table 8 and Table 9).

### 3.8. Determining Potential Risk Factors

The study found that the risk factors for RVFV seropositivity at individual livestock was sex of livestock. Female livestock were (OR: 1.74, 95% CI = 1.08–12.82) times more likely to be seropositive to RVFV than male livestock (*p* = 0.016), while areas receiving approximately 1001–1500 mm of rainfall and herd composition were risk factors at the herd level. Livestock herds in areas with rainfall amount >1600 mm were (OR: 2.239, 95% CI = 1.07–8.82) times more likely to be seropositive to RVFV than those in areas of rainfall amount of < 1000 mm at (*p* = 0.023). Further, livestock herds in areas of rainfall amount of 1001–1500 mm, were (OR: 2.470, 95% CI = 1.14–5.37) times more likely to be seropositive to RVFV than those in areas of rainfall amount of <1000 mm at (*p* = 0.022). Livestock herds that belonged to mixed species were (OR: 10.410, 95% CI = 3.04–35.59) times more likely to be seropositive to RVFV than livestock herds managed under single species (*p* = 0.001) (Table 10 and Table 11).

## 4. Discussion

This sero-epidemiological investigation reports the detection of RVFV antibodies (IgG and IgM) in apparently healthy domestic ruminants and also evaluates the risk of exposure to the virus in livestock in three ecological zones of Malawi. The current study reports RVFV seroprevalence of 21.35% in cattle, 25.68% in sheep, and 7.72% in goats, with the overall seroprevalence of 17.14%, which is comparable to the previously reported 18.13% RVFV seroprevalence in cattle from Zomba district in Malawi in 1992 [44]. The detection of IgG and IgM in this study in livestock that had no clinical signs supports the notion that RVF is endemic in sub-Saharan African countries in general, including Malawi. More importantly, these results support evidence of IEP maintenance of RVFV in domestic ruminants

The high seroprevalence in sheep (25.68%) compared with cattle (21.35%) and goats (7.72%) was likely due to the increased susceptibility of sheep to RVFV infection [60,61]. These findings are in tandem with reports from the Democratic Republic of Congo, Tanzania, and Chad where seroprevalence in sheep was higher than in cattle and goats [18,21,61,62]. The seroprevalence in sheep herds could indicate possible circulation of RVFV that caused the death of sheep in the rainy season (January–March) without clinical signs, which corroborated with prevailing fear among sheep farmers, as previously reported [43].

In addition, RVFV seropositivity was detected across all age groups of ruminant species tested, with higher prevalence in sub-adults and old-age groups compared with young animals (Table 4). The high seroprevalence in sub-adults could be due to increased activity of the group members and their high proportions in the population. Higher seroprevalence in old-age groups could be attributed to lower numbers of these age groups in the population because most adult livestock were selected for slaughter, except for the breeding stock, as previously reported [20]. Nevertheless, differences in seroprevalence between ruminant species were previously reported in other parts of the region, such as the Republic of South Africa [20,63] and Mozambique [22,33,39].

The overall difference in prevalence based on sex was reported in Chad [62] and Madagascar [11], where the higher seroprevalence in males was attributed to their roles as draft and breeding animals. Similarly, the current study observed higher seroprevalence in male than female cattle, possibly due to poor participation of male compared with female livestock. It was learned that the overall prevailing breeding management strategy preserved more cows for reproduction purposes than bulls, in a breeding ratio of approximately 1 bull to 15 or more cows. In areas dominated by indigenous cattle, they cull unwanted bulls, while in dairy production districts, artificial insemination is the predominant means of breeding [64].

The detection of IgM antibodies suggested recent virus infections in the study area, as IgM antibodies to RVFV can be detected up to two months after infection [55,57]. The circulation of RVFV during the inter-epidemic period leaves open the possibility that clinical RVF cases may have occurred undetected or may have been mistaken for other diseases, as such were not reported due to lack of public awareness [43]. The detection of IgG in other districts could be explained by increased permanent water bodies as previously reported in Tanzania and Madagascar [65,66]. Furthermore, cross-border movement could also contribute since some of the districts share boundaries with Tanzania, Mozambique, and Zambia where RVF was reported by Tshilenge et al. [6].

The significant risk factor for individual livestock was sex. Female livestock were at greater risk of RVFV infection than male livestock possibly due to higher proportions in the community, as previously reported by Nyakarahuka et al. [67]. At herd level, risk factors suggested that livestock herds living in areas with higher rainfall amounts (>1000 mm) were at greater risk of RVFV infection than livestock herds living in areas with low rainfall amount (<1000 mm), possibly due to increased favorable mosquito breeding habitats such as wetlands/dambos, as previously observed in Nigeria, Kenya, and Southern Africa [68,69,70]. In addition, livestock herds that belonged to mixed species were at greater risk of RVFV infection compared with livestock herds that belonged to single species. This could be due to increased exposure to RVFV predisposing factors such as contact with remains of neonatal death, after-birth materials, aborted/stillbirth materials (including fluids), and mosquito bites in communal grounds [43].

This study highlights the inter-epizootic maintenance of the RVFV in domestic ruminants in Malawi. The seroprevalence data from this study are vital in designing effective prevention and control strategies at national and regional levels. However, it is imperative that the role of mosquitoes, humans, and wildlife in the epidemiology of RVF in Malawi is clarified.

## 5. Conclusions

The current study updates the RVFV status in Malawi by confirming its widespread seropositivity and evidence of possible unreported active/recent infections. Animal sex, rainfall intensity, and mixed species herds are determining factors for the spread of RVFV. As RVFV is a zoonotic infection, it is necessary to conduct community sensitization and set up early warning, surveillance, and control strategies based on the identified risk factors. However, due to financial constraints, the study was not conducted in the wet and dry seasons to establish seasonal influence on RVFV circulation. The study also was unable to demonstrate the genome and the circulating strain because it failed to time the effective period of viremia state of the animals for the isolation of the virus.

## Figures and Tables

**Table 1:** RVFV seroprevalence for the livestock species and prevalence for antibody tests.

Species	Antibody Test	*n*	Reactors	Seroprevalence (%)	95% CI
Cattle	IgG	857	160	18.67	16.20–21.41
	IgM	857	23	2.68	1.79–3.99
	Overall	857	183	21.35	18.68–24.28
Goat	IgG	518	32	6.18	4.41–8.59
	IgM	518	8	1.54	0.78–3.01
	Overall	518	40	7.72	5.64–10.45
Sheep	IgG	148	24	16.22	11.14–22.99
	IgM	148	14	9.46	5.71–15.25
	Overall	148	38	25.68	19.02–33.62

*n* = Number of livestock; CI = Confidence interval; IgG = Immunoglobin G; IgM = Immunoglobin M; % = Percent.

**Table 2:** RVFV seroprevalence in ecological zones and districts.

Ecological Zones	Districts	*n*	Seroprevalence	95% CI
EZ 1	Chikwawa	228	10.96	7.54–15.68
	Nsanje	179	21.79	16.37–28.39
	Salima	202	23.76	18.42–30.09
	Mangochi	222	25.68	20.38–31.81
	Overall	831	20.34	17.68–23.27
EZ 2	Chiradzulu	151	11.26	7.15–17.29
	Thyolo	163	12.27	8.09–18.19
	Overall	314	11.78	8.53–15.99
EZ 3	Chitipa	185	23.24	17.74–29.84
	Karonga	193	6.22	3.59–10.55
	Overall	378	14.55	11.23–18.61

*n* = Number of herds; 95% CI = Confidence interval; % = Percent; EZ1 = Ecological Zone 1, EZ2 = Ecological Zone 2, EZ3 = Ecological Zone 3.

**Table 3:** Distribution of RVFV seroprevalence by the sex of animals.

Species	Sex Category	Antibody	Reactors	*n*	Seroprevalence (%)	95% CI
	Male	Overall	22	77	28.57	19.13–40.17
	Female	Overall	161	780	20.64	17.88–23.69
	Male	IgG	19	77	24.67	15.86–36.05
Cattle	Female	IgG	141	780	18.08	15.47–20.99
	Male	IgM	3	77	3.89	1.01–11.73
	Female	IgM	20	780	2.56	1.61–4.01
	Male	Overall	4	47	8.51	2.76–21.27
	Female	Overall	36	471	7.64	5.48–10.52
	Male	IgG	4	47	8.51	2.76–21.27
Goat	Female	IgG	28	471	5.94	4.05–8.58
	Male	IgM	0	47	0.00	0.00–9.41
	Female	IgM	8	471	1.69	0.79–3.45
	Male	Overall	7	18	38.89	18.26–63.86
	Female	Overall	31	130	23.85	17.00–32.27
	Male	IgG	6	18	33.33	14.35–58.84
Sheep	Female	IgG	18	130	13.84	8.63–21.26
	Male	IgM	1	18	5.55	0.29–29.37
	Female	IgM	13	130	10	5.65–16.81

*n* = Number of livestock; CI = Confidence interval; IgG = Immunoglobin G; IgM = Immunoglobin M.

**Table 4:** Distribution of RVFV seroprevalence across age groups.

Species	Age Groups (Years)	Antibody	Reactors	*n*	Seroprevalence (%)	95% CI
	<2	Overall	11	213	5.16	2.74–9.30
	2–4	Overall	97	256	37.89	31.98–44.17
	5–8	Overall	64	348	18.39	14.54–22.95
	≥9	Overall	11	40	27.50	15.14–44.13
	<2	IgG	8	213	3.85	1.75–7.53
Cattle	2–4	IgG	89	256	34.76	29.01–40.98
	5–8	IgG	54	348	15.52	11.96–19.85
	≥9	IgG	9	40	22.50	11.40–38.85
	<2	IgM	3	213	1.41	0.36–4.39
	2–4	IgM	8	256	3.12	1.46–6.29
	5–8	IgM	10	348	2.87	1.47–5.39
	≥9	IgM	2	40	5.00	0.87–18.21
	<2	Overall	6	158	3.79	1.55–8.45
	2–3	Overall	23	210	10.95	7.21–16.17
	4–5	Overall	10	136	7.35	3.78–13.45
	≥6	Overall	1	14	7.14	0.37–35.83
	<2	IgG	4	158	2.53	0.81–6.76
Goat	2–3	IgG	18	210	8.57	5.30–13.41
	4–5	IgG	9	136	6.62	3.26–12.55
	≥6	IgG	1	14	7.14	0.37–35.83
	<2	IgM	2	158	1.26	0.22–4.97
	2–3	IgM	5	210	2.38	0.87–5.77
	4–5	IgM	1	136	0.74	0.04–4.64
	≥6	IgM	0	14	0.00	0.00–26.76
	<2	Overall	3	46	6.52	1.69–18.92
	2–3	Overall	24	68	35.29	24.36–47.90
	4–5	Overall	10	32	31.25	16.74–50.14
	≥6	Overall	1	2	50.00	9.45–90.55
	<2	IgG	2	46	4.34	0.75–16.03
	2–3	IgG	11	68	16.17	8.75–27.52
Sheep	4–5	IgG	9	32	28.13	14.39–46.97
	≥6	IgG	1	2	50.00	9.45–90.55
	<2	IgM	1	46	2.17	0.11–12.96
	2–3	IgM	13	68	19.11	10.95–30.82
	4–5	IgM	1	32	3.12	0.16–17.99
	≥6	IgM	0	2	0.00	0.00–80.21

*n* = Number of livestock; CI = Confidence interval; IgG = Immunoglobin G; IgM = Immunoglobin M.

**Table 5:** RVFV herd seroprevalence across districts and species.

Factor	District	*n*	Seroprevalence (%)	95% CI
	Salima	29	44.83	26.95–64.02
	Mangochi	19	84.24	59.51–95.83
	Chikwawa	20	55.00	32.04–76.17
Districts	Nsanje	17	76.50	49.80–92.18
	Chiradzulu	80	16.13	9.36–26.55
	Thyolo	63	23.78	14.35–36.49
	Chitipa	70	42.89	31.28–55.22
	Karonga	63	14.33	7.14–25.97
	Total	361	33.24	28.18–38.11
	Salima	10	7.01	35.37–91.91
	Mangochi	12	83.32	50.88–97.06
	Chikwawa	10	30.00	8.09–64.63
	Nsanje	8	87.51	46.68–99.34
	Chiradzulu	39	28.24	15.55–45.10
Cattle	Thyolo	32	31.34	16.75–50.14
	Karonga	53	11.30	4.69–23.72
	Chitipa	60	45.00	32.33–58.31
	Salima	16	18.76	4.97–46.31
	Mangochi	5	80.00	29.88–98.95
	Chikwawa	5	60.00	17.04–92.74
	Nsanje	6	50.00	18.76–81.23
	Chiradzulu	41	4.92	0.81–17.05
Goat	Thyolo	31	16.12	6.09–34.47
	Chitipa	10	30.00	8.09–64.63
	Karonga	9	22.22	3.95–59.81
	Salima	3	100	19.79–1.00
Sheep	Mangochi	2	100	19.79–1.00
	Chikwawa	5	100	56.55–1.00
	Nsanje	3	100	39.58–1.00
	Karonga	1	100	5.46–1.00

*n =* Number of herds; CI = Confidence interval; % = Percent.

**Table 6:** Frequency and proportions of epidemiological factors at individual livestock level.

Factor	Level	Frequency	Percent (*n* = 1523)
Gender of farmer	Male	1226	80.50
	Female	297	19.50
Species	Cattle	857	56.27
	Goat	518	30.01
	Sheep	148	9.72
Sex of livestock	Male	143	9.39
	Female	1380	90.61
Ecological zones	EZ1	831	54.56
	EZ2	314	20.62
	EZ3	378	24.82
Districts	SA	202	13.26
	MH	222	14.58
	CK	228	14.97
	NE	179	11.75
	TO	163	10.70
	CZ	151	9.91
	KA	185	12.15
	CP	193	12.67
	None	19	1.25
Night shelter	Communal	862	56.60
	Private	642	42.15
Herd composition	Single species	109	7.16
	Mixed species	1414	92.84
Grazing site	Communal	1214	79.71
	Stall feeding	309	20.29
Permanent water	Swamps	634	41.53
	Swamps and rivers	889	58.47
Vegetative cover	Trees	632	41.50
	Trees and green grass	513	33.68
	Forest	378	24.82
Education level	None	69	4.53
	Primary	1010	66.32
	Secondary	412	27.05
	Tertiary	32	2.10
Routine management	No	385	25.28
	Yes	1138	74.72
Average rainfall	<1000 mm	393	25.80
	1001–1500 mm	660	43.34
	>1600 mm	470	30.86
Herd size	<25	864	56.72
	26–50	196	12.87
	≥ 51	463	30.40
RVF awareness	Yes	54	3.55
	No	1469	96.45

*n* = Number of livestock; EZ1 = Ecological Zone 1, EZ2 = Ecological Zone 2, EZ3 = Ecological Zone 3; District names: SA = Salima, MH = Mangochi, CK = Chikwawa, NE = Nsanje, TO = Thyolo, CZ = Chiradzulu, CP = Chitipa, and KA = Karonga; IgG = Immunoglobin G; IgM = Immunoglobin M.

**Table 7:** Frequency and proportions of epidemiological factors at herd level.

Factor	Level	Frequency	Percent (*n* = 361)
Gender of farmer	Male	210	58.17
	Female	151	41.83
	Goat	123	34.07
Species	Cattle	224	62.05
	Sheep	14	3.88
District	SA	29	8.03
	MH	19	5.26
	CK	20	5.54
	NE	17	4.71
	TO	63	17.45
	CZ	80	22.16
	KA	70	19.39
	CP	63	17.45
Ecological zone	EZ1	85	23.55
	EZ2	143	39.61
	EZ3	133	36.84
Night shelter	None	7	1.94
	Communal	179	49.58
	Private	175	48.48
Herd composition	Single species	69	19.11
	Mixed species	292	80.89
Grazing site	Communal	219	60.66
	Stall feeding	142	39.34
Permanent water	Swamps	56	15.51
	Swamps and rivers	305	84.49
Vegetative cover	Trees	199	55.12
	Trees and green grass	29	8.03
	Forest	133	36.84
	None	15	4.16
Education level	Primary	248	68.70
	Secondary	81	22.44
	Tertiary	17	4.71
Routine management	No	90	24.93
	Yes	271	75.07
Average rainfall	<1000 mm	28	7.76
	1001–1500 mm	258	71.47
	>1600 mm	75	20.78
Herd size	less 25	305	84.49
	26–50	18	4.99
	more than 51	38	10.53
	1–4	236	65.37
Years in farming	5–8	114	31.58
	≥ 9	11	3.05
RVF awareness	No	349	96.68
	Yes	12	3.32

*n* = Number of herds; EZ1 = Ecological Zone 1, EZ2 = Ecological Zone 2, EZ3 = Ecological Zone 3; District names: SA = Salima, MH = Mangochi, CK = Chikwawa, NE = Nsanje, TO = Thyolo, CZ = Chiradzulu, CP = Chitipa, and KA = Karonga; IgG = Immunoglobin G; IgM = Immunoglobin M.

**Table 8:** Summary of univariate regression analysis of potential risk factors and RVFV seropositivity at individual livestock level.

Potential Risk Factors	Number Tested (*n* = 1523)	Reactors (*n* = 261)	Seropositivity (%)	OR	95% CI	*p*-Value
Species (*n* = 1523)						
Cattle	857	183	21.35	Ref		
Goat	518	40	7.72	0.30	0.21–0.44	0.001 ***
Sheep	148	38	25.68	1.27	0.85–1.90	0.242 *
Sex (*n* = 1523)						
Male	142	33	23.24	Ref		
Female	1381	228	16.51	0.65	0.43–0.98	0.044 ***
Education level (*n* = 1523)						
None	69	69	100.00	Ref		
Primary	1010	111	10.99	4.55	1.41–14.64	0.011 ***
Secondary	412	72	17.47	4.96	1.51–16.20	0.008 ***
Tertiary	32	9	28.12	8.60	2.14–34.56	0.002 ***
Rainfall (*n* = 1523)						
< 1000 mm	393	75	19.08	Ref		
1001–1500 mm	660	78	11.82	0.587	0.41–0.83	0.003 ***
>1600 mm	470	110	23.40	1.33	2.96–9.81	0.044 ***
RVF awareness (*n* = 1523)						
No	1469	245	16.68	Ref		
Yes	54	16	29.63	2.10	1.15–3.83	0.015 ***
Herd composition (*n* = 1523)						
Single spp.	109	29	26.61	Ref		
Mixed spp.	1414	232	16.41	0.513	0.037–0.70	0.001 ***
Ecological zones (*n* = 1523)						
EZ3	378	55	14.86	Ref		
EZ1	831	169	20.34	1.499	1.07–2.09	0.017 ***
EZ2	314	37	11.78	0.784	0.50–1.22	0.287

*n* = Number of participants; CI = Confidence interval, Significant level < 0.05; OR = Odds ratio; *** = Significant at 0.05, considered for multivariate analysis; * = considered for multivariate analysis (cut-off *p* ≤ 0.250); Ref = reference category.

**Table 9:** Summary of test of association analysis between potential risk factors and RVFV seropositivity at herd level.

Potential Risk Factors	Number Tested (*n* = 361)	Reactors (*n* = 120)	Seropositivity (%)	OR	95% CI	*p*-Value
Species (*n* = 361)						
Cattle	224	81	36.16	Ref		
Goat	123	25	20.32	2.22	1.32–3.72	0.002 ***
Sheep	14	14	100.00	0.001	0.00–0.00	0.998
Education level (*n* = 361)						
None	15	3	20.00	Ref		
Primary	248	82	33.06	0.232	0.06–0.79	0.019 ***
Secondary	81	34	42.0	0.163	0.04–0.58	0.005 ***
Tertiary	17	1	5.88	0.23	0.01–0.37	0.014 ***
Rainfall (*n* = 361)						
< 1000 mm	28	8	28.57	Ref		
1001–1500 mm	238	73	30.37	4.925	1.44–23.35	0.024 ***
>1600 mm	75	39	52.00	9.023	1.37–17.97	0.037 ***
RVF awareness (*n* = 361)						
No	349	118	33.81	Ref		
Yes	12	2	16.67	2.587	0.55–11.99	0.225 *
Herd composition (*n* = 361)						
Mixed spp.	292	99	33.90	Ref		
Single spp.	69	21	30.43	0.353	0.02–0.80	0.022 ***
Ecological zones (*n* = 361)						
EZ3	133	39	29.32	Ref		
EZ1	85	53	62.35	6.802	3.72–12.42	0.001 ***
EZ2	143	28	19.58	1.704	0.97–2.97	0.061 *

*n* = Number of participants; CI = Confidence interval, Significant level < 0.05; OR = Odds ratio; *** = Significant at 0.05, considered for multivariate analysis; * = considered for multivariate analysis (cut-off *p* ≤ 0.250); Ref = reference category.

**Table 10:** Summary of maximum likelihood estimates for risk factors associated with RVFV seropositivity for individual livestock.

Variable	Level	OR	95% CI	*p*-Value
Sex (*n* = 1523)	Male	Ref		
	Female	1.740	1.08–12.82	0.016 ***

*** Statistically significant at *p* < 0.05; Ref = Reference category; OR = Odds Ratio, 95% CI = Confidence interval.

**Table 11:** Summary of maximum likelihood estimates for risk factors associated with RVFV seropositivity at livestock herd level.

Variable	Level	OR	95% CI	*p*-Value
Rainfall (*n* = 361)	<1000 mm	Ref		
	1001–1500 mm	2.475	1.14–5.37	0.022 ***
	>1600 mm	2.239	1.07–8.83	0.023 ***
Herd composition (*n* = 361)	Single spp.	Ref		
	Mixed spp.	10.410	3.04–35.59	0.001 ***

*** = Significant at 0.05; OR = Odds ratio; CI = Confidence interval; Significant level < 0.05; Ref = Reference category.

## Data Availability

All data have been provided in the article and/or as supplement documents.

## Supplementary Materials

The following are available online at https://www.mdpi.com/article/10.3390/pathogens11111349/s1, Table S1. Optical Density Wavelength Readings for the RVFV IgG Antibodies, Table S2. Optical density wavelength readings for the RVFV IgM Antibodies, Table S3. Distribution of serum samples across the ecological zones and antibody seropositive results at individual livestock level, Table S4. Distribution of serum samples across the ecological zones and districts and antibody seropositive results at herd level.
