# Supplementary material for: Seroprevalence and Associated Risk Factors of Rift Valley Fever in Livestock from Three Ecological Zones of Malawi
Source: Pathogens. 2022 Nov 14;11(11):1349. doi: 10.3390/pathogens11111349 (PMC9698272; doi:10.3390/pathogens11111349)
Supplement: Supplementary file 1 [file pathogens-11-01349-s001.zip › pathogens-1982201-supplimentary.pdf]

Supplementary Material

Contains:

1. Table S1: Optical Density Wavelength Readings for the RVFV IgG Antibodies
2. Table S2: Optical density wavelength readings for the RVFV IgM Antibodies
3. Table S3: Distribution of serum samples across the ecological zones and antibody seropositive results at individual livestock level
4. Table S4: Distribution of serum samples across the ecological zones and districts and antibody seropositive results at herd level

OPTICAL DENSITY READINGS FOR THE SERUM SAMPLES (IgG and IgM Tests)

Table S1. Optical Density Wavelength Readings for the RVFV IgG Antibodies.

No.	Sample ID	O.D Reading	S/N %	No.	Sample ID	O.D Reading	S/N %
1	6	0.596	31	24	136	0.701	38
2	19	0.057	5	25	137	0.047	8
3	26	0.194	16	26	138	0.106	8
4	27	0.739	39	27	157	0.391	31
5	29	0.696	37	28	170	0.121	10
6	47	0.296	24	29	195	0.096	8
7	52	0.693	36	30	124	0.357	29
8	54	0.681	36	31	142	0.136	7
9	55	0.06	15	32	143	0.348	28
10	56	0.72	38	33	162	0.625	33
11	68	0.573	30	34	165	0.233	15
12	70	0.259	21	35	175	0.388	31
13	71	0.262	14	36	176	0.793	33
14	83	0.471	28	37	217	0.108	4
15	102	0.107	9	38	218	0.11	4
16	103	0.709	37	39	219	0.693	28
17	105	0.394	32	40	235	0.127	5
18	106	0.063	5	41	269	0.085	3
19	108	0.064	5	42	270	0.526	21
20	115	0.465	28	43	283	0.879	35
21	119	0.487	26	44	302	0.981	39
22	125	0.722	39	45	310	0.174	7
23	128	0.485	25	46	382	0.126	5
47	387	0.297	12	70	535	0.173	9
48	395	0.592	23	71	536	0.126	7
49	424	0.116	5	72	537	0.199	11
50	425	0.142	6	73	538	0.087	5
51	426	0.687	27	74	539	0.454	25
52	436	1.01	40	75	540	0.098	5
53	437	0.876	35	76	547	0.541	30
54	866	0.36	14	77	552	0.393	21
55	489	0.101	6	78	553	0.218	12
56	490	0.085	5	79	566	0.071	4
57	492	0.514	28	80	571	0.077	4
58	503	0.1	5	81	572	0.166	9
59	508	0.195	11	82	573	0.063	3
60	513	0.513	28	83	574	0.279	15
61	516	0.517	28	84	575	0.451	25
62	520	0.274	15	85	576	0.733	40
63	522	0.121	7	86	577	0.352	19
64	526	0.136	7	87	586	0.34	19
65	527	0.147	9	88	590	0.252	14
66	528	0.157	9	89	591	0.136	7
67	530	0.303	17	90	593	0.183	10
68	532	0.09	5	91	596	0.066	5
69	533	0.126	7	92	598	0.19	15
93	621	0.42	34	118	940	0.07	4
94	657	0.059	5	119	941	0.143	9
95	658	0.352	28	120	952	0.388	25
96	664	0.322	25	121	958	0.077	5
97	665	0.056	5	122	961	0.529	33
98	666	0.09	7	123	963	0.457	29
99	667	0.281	23	124	966	0.093	6
100	668	0.057	5	125	968	0.632	40

101	669	0.38	31	126	969	0.083	5
102	678	0.308	25	127	973	0.288	18
103	679	0.475	38	128	974	0.064	4
104	680	0.057	5	129	977	0.387	4
105	681	0.209	17	130	978	0.066	4
106	685	0.326	26	131	981	0.379	24
107	686	0.382	31	132	990	0.081	5
108	697	0.455	37	133	994	0.048	3
109	809	0.052	4	134	996	0.284	18
110	820	0.095	8	135	997	0.052	3
111	821	0.076	6	136	1007	0.058	4
112	829	0.783	36	137	1009	0.058	4
113	836	0.755	34	138	1012	0.438	28
114	834	0.85	39	139	1013	0.059	4
115	918	0.136	9	140	1015	0.387	24
116	919	0.393	25	141	1032	0.093	6
117	920	0.369	23	142	1064	0.068	4
143	1073	0.055	3	167	729	0.656	37.8
144	1074	0.349	22	168	739	0.064	3.7
145	1342	0.069	4	169	740	0.308	17.8
146	1368	0.073	5	170	747	0.561	32.3
147	1374	0.056	4	171	754	0.07	4.0
148	1378	0.053	3	172	759	0.128	7.4
149	1379	0.474	30	173	760	0.651	37.5
150	1388	0.054	3	174	766	0.072	4.1
151	1389	0.312	20	175	767	0.057	3.3
152	1390	0.612	39	176	769	0.134	7.7
153	1452	0.34	24	177	775	0.571	32.9
154	1462	0.063	4	178	776	0.647	37.3
155	1463	0.344	24	179	778	0.214	12.3
156	1510	0.063	4	180	782	0.428	24.7
157	1512	0.568	40	181	783	0.076	4.4
158	1518	0.06	4	182	788	0.068	3.9
159	1519	0.611	6	183	790	0.648	37.3
160	1520	0.078	6	184	791	0.091	5.2
161	1522	0.056	4	185	794	0.06	3.5
162	1523	0.305	22	186	796	0.087	5.0
163	715	0.075	4.3	187	800	0.087	5.0
164	720	0.254	14.6	188	801	0.095	5.5
165	727	0.109	6.3	189	1106	0.174	10.0
166	728	0.064	3.7	190	1107	0.3	17.3
191	1154	0.072	4.1	214	543	0.683	36
192	1196	0.067	3.9	215	846	0.624	33
193	1208	0.215	12.4	216	1109	0.089	5
194	1232	0.635	36.6				
195	1249	0.137	7.9				
196	1258	0.061	3.5				
197	1259	0.145	8.4				
198	1260	0.376	21.7				
199	1262	0.084	4.8				
200	1263	0.069	4.0				
201	1264	0.075	4.3				
202	1265	0.344	19.8				
203	1266	0.615	35.4				
204	1267	0.064	3.7				
205	1268	0.391	22.5				
206	1237	0.176	10.1				
207	773	0.42	37				
208	1395	0.14	12				

209	448	0.106	6
210	449	0.09	5
211	450	0.137	7
212	505	0.749	40
213	541	0.742	40

No. = Serial number; Sample ID = Sample Identity; O.D reading = Optical Density reading; S/N%= Suspect or Negative Value Percentage.

Table S2. Optical density wavelength readings for the RVFV IgM Antibodies.

No.	Sample ID	O.D Reading	S/N %	No.	Sample ID	O.D Reading	S/N %
1	6	0.273	69	36	992	0.229	51
2	57	0.253	53	37	1009	0.229	51
3	104	0.295	62	38	1041	0.294	66
4	112	0.239	56	39	1075	0.333	51
5	150	0.229	51	40	1134	0.346	57
6	156	0.229	51	41	1139	0.319	53
7	174	0.231	52	42	1140	0.352	58
8	179	0.284	73	43	1141	0.321	53
9	189	0.264	76	44	1142	0.312	51
10	211	0.259	72	45	1146	0.335	55
11	217	0.299	76				
12	219	0.262	73				
13	221	0.268	76				
14	263	0.295	82				
15	281	0.297	87				
16	303	0.224	66				
17	336	0.231	81				
18	389	0.287	54				
19	421	0.224	77				
20	473	0.283	78				
21	511	0.334	91				
22	640	0.301	59				
23	762	0.274	58				
24	766	0.247	52				
25	777	0.388	87				
26	779	0.373	67				
27	781	0.337	76				
28	785	0.334	70				
29	786	0.373	78				
30	799	0.344	54				
31	800	0.238	50				
32	821	0.253	53				
33	831	0.295	62				
34	916	0.284	73				
35	956	0.231	52				

No. = Serial number; Sample ID = Sample Identity; O.D reading = Optical Density reading; S/N%= Suspect or Negative Value Percentage.

Table S3. Distribution of serum samples across the ecological zones and antibody seropositive results at individual livestock level.

**Ecological Zones**						EZ 1						EZ 2				EZ 3		Total sampled
**Number of Animals Sampled**						831						314				378		1523
	**District**	**SA**		**MH**		**CK**		**NE**		**TO**		**CZ**		**CP**		**KA**		
**Livestock Species**	**Sampled**	IgG	IgM	IgG	IgM	IgG	IgM	IgG	IgM	IgG	IgM	IgG	IgM	IgG	IgM	IgG	IgM	Total reactors
**Cattle**	857	32	1	37	8	5	1	21	2	12	2	14	1	33	7	6	1	183
**Goat**	518	7	0	9	0	2	2	7	1	4	2	1	1	0	3	2	0	40
**Sheep**	148	8	0	3	0	2	13	8	0	0	0	0	0	0	0	3	0	38
Total	1523	47	1	49	8	9	16	36	3	16	4	15	2	33	10	11	1	261
EZ total reactors						169						37				55		

IgG= Immunoglobin G; IgM= Immunoglobin M; EZ=Ecological zone; EZ 1= Ecological zone 1, EZ 2= Ecological zone 2, EZ 3= Ecological zone 3; Ditrict names: SA= Salima, MH=Mangochi, CK=Chikwawa, NE=Nsanje, TO= Thyolo, CZ=Chiradzulu, CP= Chitipa, and KA= Karonga.

Table S4. Distribution of serum samples across the ecological zones and districts and antibody seropositive results at herd level.

		EZ 1								EZ 2				EZ 3				Total sampled
EZ herds Sampled		85								143				133				361
	**Districts**	**SA**		**MH**		**CK**		**NE**		**TO**		**CZ**		**CP**		**KA**		
Number of sampled herds		29		19		20		17		63		80		70		63		
		IgG	IgM	IgG	IgM	IgG	IgM	IgG	IgM	IgG	IgM	IgG	IgM	IgG	IgM	IgG	IgM	
Antibody tests Livestock species	Sampled																	Total reactors
**Cattle**	224	6	1	6	4	2	1	5	2	8	2	10	1	21	6	5	1	81
**Goat**	123	3	0	4	0	1	2	3	0	3	2	1	1	0	3	2	0	25
**Sheep**	14	3	0	2	0	1	4	2	1	0	0	0	0	0	0	1	0	14
District total		13		16		11		13		15		13		30		9		120
EZ total reactors		29								52				39				

n = number; IgG= Immunoglobin G; IgM= Immunoglobin M; EZ=Ecological zone; EZ 1= Ecological zone 1, EZ 2= Ecological zone 2, EZ 3= Ecological zone 3; District names: SA= Salima, MH=Mangochi, CK=Chikwawa, NE=Nsanje, TO= Thyolo, CZ=Chiradzulu, CP= Chitipa, and KA= Karonga.
